# Experiences and ways PLWHA deal with their lives

**DOI:** 10.4314/ahs.v21i4.15

**Published:** 2021-12

**Authors:** Anthony Kiwanuka, Eddy Walakira, David Kaawa-Mafigiri

**Affiliations:** 1 Makerere University College of Humanities and Social Sciences, Department of Social Work and Social Administration; 2 Uganda Christian University, Faculty of Social Sciences, Department of Social work and Social Work Administration

**Keywords:** Health Services, seeking behavior, persons living with HIV

## Abstract

The issue of HIV and AIDS and people living with HIV and AIDS is very sensitive which needs great attention. The population of infected individuals seems not to seek help and health services due to their illness. The barriers which prevent this population experience the meaning of social construction of their illness. The main objective of this study is to understand the day-to-day lives and the ways PLWHA deals with their lives. Qualitative method and participatory action research were used to gather information. The study found out that PLWHA have much to fight for in the complex and frightening areas of HIV research and treatment. Though there have been lacking scientific skills, all PLWHA can bring unique experiences and perspectives to an open discussion. The study concluded that facing the challenges of working with HIV and AIDS -individuals; consider the participatory research approach because it can reach out to individuals, groups and organizations. It was recommended that the ultimate goals of AIDS treatment and research activism are to accelerate scientific research knowledge that contributes towards cure, and to win an early widespread access to treatment for everyone infected with HIV. Developing a well-designed research can help PLWHA learn how to ask themselves different questions and ask if the medical and research establishments will address their concerns.

## Introduction

It is clear that PLWHA have much to fight for in the complex and frightening areas of HIV research and treatment. Though there have been lacking scientific skills, all PLWHA can bring unique experiences and perspectives to an open discussion. HIV-infected individuals know which issues are important to them and their loved ones, what doubts and fears they have about treatment and how it is or is not working in their bodies^1^. PLWHA know the reservations they and other infected individuals have about being research subjects and what might answer these reservations. Developing a well-designed research can help PLWHA learn how to ask themselves different questions and ask if the medical and research establishments will address their concerns.

In contemporary social work practice, social workers expect to meet challenges with people living with HIV and AIDS (PLWHA) whose experience of illness is due to the social construction of meaning around HIV and AIDS. Practically, HIV and AIDS is a very sensitive issue that requires great attention when social work intervention is to succeed. There is a dire need to begin developing the knowledge and skills necessary to work effectively with this population. It is from this perspective; we need to explore the unique experiences of the disease and the social meaning of HIV and AIDS in particular. PLWHA can be traumatized right from the time they are found HIV -positive. This trauma is connected and associated to stigma that goes along with the disease and include negative words and some ill spoken words that are used by people in their respective communities. The naming of the disease, most of which have negative implications; such as physical, verbal abuse and neglect, physical violence, sexual exploitation and abuse by men and women, victimization in relationships; where some women and men face rape by HIV-positive husbands and women who know that they are HIV-positive^2^. Clinical research needs to inquire about issues concerning PLWHA and their unique experiences.

## Problem Statement

Little attention seems to have been paid to constructions of HIV and AIDS in the society's context and to the processes by which such constructions are experienced, understood, reacted to and perhaps, reconstructed through social and interactions^3^. People living with HIV and AIDS illness, experiences and HIV and AIDS as a social construct is understood in the context of their individual daily encounters. Despite the knowledge of HIV and AIDS, PLWHA's perceptions and responses to this disease is dully influenced by their experiences of interacting with others for instance, their school mates, families, friends, co-workers and teammates in clubs, companies or associations^3^. PLWHA may experience social problems associated with the disease. The negative attitudes towards infected individuals in the families, schools, communities might be some of the barriers that hinder how PLWHA interact in a free environment that leads to the existence of stigma and discrimination in society. Capturing information on how people living with HIV and AIDS experience the illness helps the clinical researcher get either prepared or not to make effective intervention with this population. Therefore, research is the only way to obtain adequate information with evidence to practice effectively. Questioning why this is so, the study sought to examine the day-to-day lives and the ways PLWHA deals with their lives.

## Purpose

The purpose of the study was to understand the day-to-day lives and the ways PLWHA deals with their lives

## Objective

To assess PLWHA with unique experience of illness and social construction of meaning of HIV and AIDS

## Review of Related Literature

### PLWHA with unique experience of illness and social construction of meaning of HIV and AIDs

The unique experience of illness and social construction of HIV and AIDS should be phenomenological explored because it is one of the goals to understand human science from the view of the human world. PLWHA live in the world and have various experiences in everyday situations and relationships, so they are in a position to tell of their lived experiences with HIV and AIDS. Understanding the social construction of HIV and AIDS, the individuals infected and who have lived through those experiences get involved in their immediate pre-reflective consciousness of life; a reflective or self -given awareness that is, being aware, and unaware of itself according to^4^. It is believed that experiences “lived” have a temporal structure, for example, PLWHA reflecting on how the infection, affected their lives in day-to-day situations.

HIV and AIDS cannot be understood in its immediate manifestation but only reflectively as past experience. The reason for looking at this population (PLWHA) phenomenologically is to access their experiences and their reflections on the HIV and AIDS epidemic. Such experiences can help social work researchers to be able to come up with a proper understanding of the deeper meaning of an aspect of human experience in the context of the whole human experience of PLWHA^5^. This helps the clinical social work practitioner to understand the scientific and ethical methods, knowledge building and knowledge for practice. The findings may also assist the clinical researcher to apply the same knowledge to use research evidence to inform practice and inquiry^6^. People living with HIV and AIDS can describe their illness from the common factors in mental health problems that change the way they function and perform in their communities. They are affected in ways of thinking, feeling and behaving, and their functioning at school, work and in relationships is impacted^7^. Living with the virus is a mental challenge and a near universal experience when one reads “you have HIV.” The individual's definition of his/her self-image changes immediately, regardless of culture, age, race, social or economic, geography or relationship status^8^. For some it is a surprise, for others it is a confirmation of what s/he suspected. In most cases this is an isolating and a daunting situation with significant psychological ramifications^8^. They may feel that they cannot cope effectively with life's difficulties and experiences such as; job loss and stress, death of a loved one, ending a relationship, financial problems and family difficulties.

People Living with HIV and AIDS may lose hope, feel unworthy to live and give up taking care of themselves physically, for example, seeking help and taking HIV medication. It might be a problem for people living with HIV and AIDS making healthy choices about doing exercises, getting enough sleep and not having unprotected sex^7^. They may have trouble in managing their feelings and unhealthy thinking patterns which are psychological factors that lead to stressful lives or psychosocial stressors. They may be faced with trauma, sexual and physical abuse neglect and HIV infection. They may be stressed and this can contribute to their mental health problems such as; loss of support which results in isolation, difficulty in coping with HIV and AIDS as a disease or illness, trouble getting the services they need and hardship in telling others that they are HIV-positive^7^. Due to changes in their physical appearance and abilities due to HIV and AIDS, they may feel empty and neglected or stigmatized and discriminated against.

PLWHA have different behaviors, thoughts and feelings that indicate the need for health care and social services. They may experience many of the symptoms differently in their lives such as feelings of sadness, an overwhelming sense of pessimism, feeling they want to hurt themselves and thinking that they would be better off dead^7^. They may be found themselves in a situation of being unable to have fun or derive pleasure from activities that provided pleasure in the past, racing thoughts, inability get rid of persistent worries, frequent night mares, sleeping too much, use of alcohol or drugs that is interference in their life style and having changes in appetite and eating habits^7^, ^8^. These symptoms and many others which are not mentioned they can become a problem when they don't go away and are occurring at the same time. Many of the people living with HIV and AIDS suffer for a long time periods and they can live positive life if treatment is provided but more than half of the HIV-infected individuals who need treatment do not seek or receive HIV and AIDS treatment and services^7^.

The society where infected individuals are found with above the mentioned symptoms links the PLWHA with HIV and AIDS and; connects them to immorality and other negative characteristics and finally behavioral consequences of stigma which might describe isolation, distancing and discrimination in taking care of them^8^. We consider the society with its diversity that plays a major role in a crucial way and duly contributes to the strength of distancing the reactions and discrimination of PLWHA^5^. This is due to the fact that cultures in society are the basis of constructing the meaning of HIV and AIDS based on beliefs about contamination, sexuality and religion. People in society identify PLWHA as negligent, sex workers, irresponsible and many other stigmatizing responses that define the problem of HIV and AIDS and then link PLWHA to HIV and AIDS.

## Methods

The researcher used a study population and sampling of twenty PLWHA and conduct one to one interview that include semi-structured in-depth questionnaires. PLWHA two clinical centers were conveniently sampled to participate in the study. The participants were assured of confidentiality and asked to tell their stories; this assisted those who were uncomfortable talking in a group, or wishing to keep their stories confidential. Four Participatory action research group discussions were used to get more information and generate knowledge concerning HIV and AIDS dilemmas and difficulties that prevail by not seeking services. The researcher asked participants to get into groups of eight to ten people to conduct the discussions on their own issues. Observation was one of the methods that will be used during interviews, for example, observing participant's non-verbal behavior such as emotional expressions and body language. In the observation during the study, field notes and reflective documents were also considered. Review of documents concerning people living with HIV and AIDS enabled the researcher to create knowledge that can be used in day-to-day social work practice^9^.

By using PAR with people living with HIV and AIDS was the starting point which is helpful to the clinical researcher because: PAR will provide a way of exploring PLWHA participants' experiences and working with them to address the issues that they choose to work on or prioritize themselves^10^. It helped in the process of planning issues that need to be addressed related to the HIV and AIDS barriers in a sustainable way.

In addition, getting knowledge for practice and to address the community concerns of PLWHA was to continue using participatory research on community health systems for HIV treatment and support^11^. The researcher used ten key informants' interviews, review of the existing literature concerning HIV and AIDS and its consequences, media, stakeholder discussions with NGOs, and community representatives to obtain deeper understanding of HIV and AIDS- related information.

### Study design

Qualitative research was conducted in order to obtain in-depth information from the PLWHA. Since this approach has the natural setting as the direct source of data and the researcher. This has a lot to do with getting considerable time in communities and other sources where information can be obtained directly^11^. The issue of HIV and AIDS related barriers could be fully understood and interpreted in an acceptable manner. From this perspective, the study was fully concerned with the description of issues that prevent PLWHA from seeking HIV treatment in their context, i.e., how they feel, observed in their settings and actions they can be understood. Dealing with qualitative research was imperative because; the study did not focus at the outcomes of this research but concerned with the process how HIV and AIDS affect the peoples' lives^9^, ^12^. In addition, the study looked at the meaning which is the essential concern of qualitative so as to capture perspectives accurately since data analysis was inductive. The study is termed qualitative using participatory action research when the main philosophy of research is pragmatism^5^, ^10^.

### Data Analysis and Processing

#### Transcription

Kansiime, 13, as cited in (Kvale,(1996), transcription in itself is an interpretative process. Since the informant's experiences of different phenomena are reported according to the phenomenological tradition, the ambition has been not to make interpretations in the collection and transcriptions of interviews. The interviews were, as mentioned above, verbatim transcribed.

The data was analyzed qualitatively in line with the key themes of the study using content and thematic analysis techniques. The first step in the analysis of the data consisted of reviewing and categorizing the textual data under different themes that were of interest in the study. Coding of data collected begun immediately after the interviews had been transcribed. This process involved working with the interview data, organizing it, breaking it into manageable units, synthesizing it, and searching for patterns to discover what was important for the study. Analysis begun with the identification of themes emerged from the raw data, a process sometimes referred to as open coding. During open coding, the researcher identified and tentatively named the conceptual categories into which the phenomena observed were grouped.

### Research Ethical Considerations

Generally, there are ethical principles for social research of which a few are prescribed here:^13^ mention that some of the following ethical principles:

Given the sensitivity of the subject under study, ethical issues related to the research and participants were considered carefully. No reference was given to specific identities of the respondents/participants. Informed consent from participants and agencies were obtained. This informed consent assured the confidentiality of their responses held under lock-and-key and only used for the purposes of this study. Furthermore, informed consent emphasized that this study was entirely voluntary and that all participants had the right to withdraw from the study any time or to not answer a particular question.

Confidentiality was assured and anonymity was insured, because contact with participants was necessary. Triangulation of chart data with interview data was undertaken. Before all interviews the participants had to sign a consent form. No participant was subjected to prejudices, stereotypes or biases, either directly or by casual or indirect suggestion, by the research team.

### Study findings

The study found out that PLWHA have much to fight for in the complex and frightening areas of HIV research and treatment. Though there have been lacking scientific skills, all PLWHA can bring unique experiences and perspectives to an open discussion. HIV-infected individuals know which issues are important to them and their loved ones, what doubts and fears they have about treatment and how it is or is not working in their bodies. PLWHA know the reservations they and other infected individuals have about being research subjects and what might answer these reservations. In addition, the research on community systems is effective to understand PLWHA in a ‘community’ because it means a group of people who have a shared relationship and interest; therefore, the researcher will use PAR tools in the field.

## Discussion

The researcher used discussion, market place and leaping blocks in order to review the evidence to assess the opportunities and mechanisms to enhance practitioners and overcome priority blocks to access. Using problem tree, ranking and scoring, and discussions, will help to identify for key social groups the priority factors that affect availability, access, acceptability, uptake, quality of care in and adherence to the services for HIV prevention, treatment and care^14^. By using the approach of participatory, we are aiming at getting relevant information which can be based on to deliver effective services in our practice. Relevance needs to be built in both during the design of the research and afterwards when useful ideas are being developed from it. It is true that translation of issues from research studies is significantly reduced if the research has at its spirit/heart knowledge and understanding of the practice that it is trying to improve, for example, treatment of HIV and AIDS patients^11^, ^15^.

## Conclusions

Facing the challenges of working with HIV and AIDS -individuals, consider the participatory research approach because it can reach out to individuals, groups and organizations, on different languages in order to develop a response to the reflection and implementation on a new outreach strategy of treating HIV and AIDS. PAR assumes that the types of knowledge we collect from this approach are valid and in fact that research should be conceptualized broadly to include: interactive knowledge which is based on connections and relationships between people, instrumental knowledge which is produced through research based on a positivistic paradigm though it is argued that it is not sufficient as a means to understand the real world, and critical knowledge which is raised from a process of reflection and action in attempting to resolve human problems^14^.

## Recommendations

It was recommended that the ultimate goals of AIDS treatment and research activism are to accelerate scientific research knowledge that contributes towards cure, and to win an early widespread access to treatment for everyone infected with HIV. Developing a well-designed research can help PLWHA learn how to ask themselves different questions and ask if the medical and research establishments will address their concerns^15^. Researchers have to use and coordinate with community clinic health care providers who are working in the primary health care settings. This will help the researcher to mention the study to people visiting the health care clinic and have the opportunity of inviting them to take a copy the study information statement, consent form and an envelope which is addressed and stamped that must be returned if they will be willing to participate in the study. The researcher has to issue the contact and request participants to sign and return the consent form if interested in participating in the study.

To empower PLWHA, there is need to involve them in research using participatory action research methods because this approach is purposely to empower them. It is through empowering this population; knowledge and action produce useful information and strategies to groups of people concerned. It encourages people to construct and utilize their own knowledge for empowerment. The approach promotes collaboration all through the research process. Thus, participatory action research is appropriate to be used with People living with HIV and AIDS in study that stresses/emphasizes empowering systems of various sizes, from individuals to the whole communities. It is clearly observed that, individuals or people understudy for example, PLWHA participate more actively with the researcher all through the research process^16^. That is to say, from the beginning of research design, to the end of the research where presentation and dissemination of the findings are dealt with.
